# Editorial: Allogenic hematopoietic cell transplant in hematological malignancies: controversies and perspective

**DOI:** 10.3389/fonc.2025.1582751

**Published:** 2025-03-19

**Authors:** Salvatore Leotta, Sabrina Giammarco, Jacopo Mariotti

**Affiliations:** ^1^ Azienda Ospedaliero-Universitaria Policlinico “G-Rodolico”- San Marco - Catania, Catania, Italy; ^2^ Dipartimento di Scienze di Laboratorio e Infettivologiche, Agostino Gemelli University Policlinic Istituto di Ricovero e Cura a Carattere Scientifico (IRCCS), Rome, Italy; ^3^ Humanitas Cancer Center, Humanitas Research Hospital, Rozzano, Italy

**Keywords:** hematologic malignancies, acute leukemia, controversies, perspectives, hematopoietic (stem cell) transplant (HSCT)

The Allogeneic Hematopoietic Stem Cell Transplantation (HSCT) represents the unique chance of cure for patients affected by some hematologic malignancies and in particular for acute leukemias, myelodysplasia (MDS) and myelofibrosis. Several unmet needs are still on the table and are object of research.

First, transplant indications. The novel classification of acute leukemias, derived from a refinement of the knowledge of the bio-molecular patterns underlining the hematologic neoplasms, allowed a more precise risk-stratification (1, 2). Based on such stratification expert panels have identified high risk-categories for which the transplant approach in the front-line setting is mandatory (3). By converse for low and intermediate risk-categories the HSCT in 1^st^ complete remission is more controversial and evaluation of minimal residual disease (MRD) in this setting plays a crucial role (4, 5).

Also for myelofibrosis and MDS the improvements in knowledge of the pathogenetic patterns refined the prognosis and the transplant’s indications (6, 7). For multiple myeloma and lymphoma, given the advent of the bi-specific antibodies and CAR-T, the perspective of HSCT has become increasingly distant and remains confined to settings such as plasma cell leukemia, relapsed/refractory Hodgkin lymphoma and some T-cell non-Hodgkin lymphomas.

Second, If by one hand, HSCT can cure a relevant proportion of patients, for about half of the transplanted patients relapse of the hematological disease and treatment-related mortality are the main causes of transplant-failure.

In the last two decades significant improvements in conditioning regimens, graft-versus host disease (GVHD) prophylaxis, management of infections and maintenance therapies have increased the opportunity to perform transplantation for an even greater number of patients by reducing transplant-related mortality (TRM) and by preventing relapse.

The introduction of the novel reduced-toxicity conditionings (8, 9) and novel GVHD-prophylaxis regimens (10–12) contributed to reduce the TRM and to extend the transplant to an increasingly large population both in terms of age, co-morbidities and absence of an HLA fully-matched donor.

The introduction of the busulfan-fludarabine regimens (8) and of treosulfan (9) have reduced the impact of the conditioning on non-hematological toxicity and extended the HSCT up to 70 years-old patients or higher. Furthermore, different combinations of alkylating drugs and dosages generates a series of reduced-intensity conditionings (RIC). The RIC regimens, if by one hand are associated with lower TRM, on the other hand correlate with a greater risk of relapse (13) and experts recommend their use in an individualized approach that takes into account age, fitness and relapse-risk (14).

The application of the T-cell-replete HSCT based on the use of post-HSCT cyclophosphamide (PTCy) has made possible to extend the haploidentical transplantation in favor of a greater number of patients who lacks an HLA-compatible donor. Furthermore, also centers that do not perform expensive *ex vivo* T-depletion may perform such procedure.

Nowadays, the results of the T-replete transplantation with PTCy from haploidentical donors are comparable with those of HSCT from unrelated and related HLA-matched donors (10–12). PTCy has been proposed as a novel GVHD-Prophylaxis regimen also for transplants from HLA-compatible donors as an alternative to antithymocyte globuline (ATG) or anti-T- lymphocyte globulin (ATLG) that, together with calcineurin-Inhibitors and methotrexate, have been for years a cornerstone of GVHD-prophylaxis (15).

In patients with donor-specific anti-HLA antibodies strategies to overcome the donor-sensitization by combining immunosuppressive therapy and plasma-exchange allowed to achieve engraftment also in such challenging situation (16).

The scenario of the pre-remissional treatment has been enriched with novel targeted drugs such as hypomethylating agents and venetoclax that have made possible for elderly patient (> 60 years old) unfit for intensive chemotherapy, to achieve complete remission and HSCT (17). Other classes of targeted drugs, such as tyrosine-kinase inhibitors and blinatumomab have increased the number of patients who reach a deeper remission before transplantation (18–21).

At the same time the introduction of the targeted therapies improved the transplant outcome when applied as post-transplant maintenance and by synergizing with the Graft Versus Leukemia effect (22–27).

The GVHD, historically considered an “orphan disease”, in the past ten years has been the subject of clinical trials that led to the approval of new agents such as ruxolitinib (FDA and EMA-approved for acute and chronic steroid-refractory GVHD) (28–29), ibrutinib (30) and belumosudil (31) (FDA-approved as second and third-line for chronic GVHD, respectively).

As regard to acute GVHD-prophylaxis, recently new drugs have undergone to investigation: vedolizumab (32), abatacept (33), fecal transplantation (34), begelomab (35), α1 antitrypsin (AAT) (36). A phase 2/3 study is evaluating the AAT as prophylaxis of acute GVHD (NCT03805789).

In regard of chronic GVHD, axatilimab, a CSF-R inhibitor, is under investigation in steroid-refractory chronic GVHD alone or in combination with Extracorporeal Photopheresis (NCT06821542, NCT06663722) following the promising results of a phase 1/2 study (37). Other studies are ongoing to investigate axatilimab as first line therapy in combination with other agents (steroids or ruxolitinib) (NCT06388564, NCT06585774).

Another line of research focuses on the use of anti-GVHD cellular therapies and the potential target represented by the T-regulatory lymphocytes (38).

Moreover, growing evidence supports the contribution of loss of the gut microbiome diversity and of the endothelial dysfunction to transplant morbidity, justifying studies aimed at developing novel therapies in these fields of application (39, 40).

The transplantation is the platform on which to add strategies aimed at preventing relapse. Novel studies evaluated the employment of post-transplant immunotherapy. Such interventions may be represented by conventional T-cells (prophylactic or pre-emptive DLI) (41), by selected subsets of T-cells (such as CD45RA-) (42) or by modified effector cells (either NK cells or T-cells redirected against leukemia antigens) (43). Results of these studies although promising, require further research.

Based on these assumptions we propose a Research Topic dedicated to HSCT. The purposes of the research-topic are:

providing transplant-physicians and hematologists with a description of the current “state of the art” in some particular settings such as HSCT from alternative donors, transplantation in multiple myeloma and primary myelofibrosis.

Furnishing experience about some topics such as transplantation from AB0- mismatched donors, impact of donor-parity on outcome of HSCT, transplantation in patients with immunological sensitization against the donor, post-transplant maintenance.

Specialists in the subjects selected and reviewed the Research Topics and we hope that it can be a valid tool to support the reader in improving the knowledge and the clinical practice.
